# Novelty and learning in cognitive control: evidence from the Simon task

**DOI:** 10.1007/s00426-023-01813-z

**Published:** 2023-03-31

**Authors:** Andrea De Cesarei, Stefania D’Ascenzo, Roberto Nicoletti, Maurizio Codispoti

**Affiliations:** 1 Department of Psychology, Alma Mater Studiorum University of Bologna, Viale Berti Pichat 5, 40127, Bologna, Italy; 2 Department of Philosophy and Communication Studies, University of Bologna, Bologna, Italy

## Abstract

While information that is associated with inappropriate responses can interfere with an ongoing task and be detrimental to performance, cognitive control mechanisms and specific contextual conditions can alleviate interference from unwanted information. In the spatial correspondence (Simon) task, interference has been consistently shown to be reduced by spatial non-correspondence in the previous trial (i.e., correspondence sequence effect, CSE); however the mechanisms supporting this sequential effect are not well understood. Here we investigated the role of novelty and trial-to-trial changes in stimulus and response features in a Simon task, observing similar modulation of CSE for novel and non-novel stimulus changes. However, changing the response modality from trial to trial dampened CSE, and this dampening was more pronounced when the probability of switch trials was higher, suggesting a role for long-term learning. The results are consistent with recent accounts, which indicate that spatial interference can be prevented by cognitive control mechanisms triggered by learned bindings.

**Supplementary Information:**

The online version contains supplementary material available at 10.1007/s00426-023-01813-z.

Achieving one’s goals entails shielding goal-oriented processing from interference, i.e., from the effects of information associated with inappropriate responses. In several tasks, it has been well-documented that task-irrelevant stimuli interfere with goal-related activity, eventually resulting in detrimental effects on performance (i.e., Flanker task, Eriksen & Eriksen, 1974; color-naming task, Stroop, 1935; spatial correspondence task, Simon & Rudell, 1967). In studies examining spatial correspondence (Simon paradigm; Simon & Rudell, 1967), participants view lateralized stimuli, and respond to a non-spatial stimulus feature (e.g., color) by using lateralized response keys (e.g., a left key for the red stimuli and a right key for the blue ones). Faster and more accurate responses are observed when the stimulus and the response position correspond spatially (e.g., both on the right, or on the left) as opposed to when they do not (e.g., stimulus on the right and response on the left, or viceversa). We will refer to this effect as correspondence effect (CE). In addition to behavioral performance, some studies have focused on the impact of spatial correspondence on pupil dilation (PD), which is modulated by a number of factors including arousal through activation of the locus coeruleus-noradrenergic system (Bradley et al., 2008; Reimer et al., 2014; van der Wel & van Steenbergen, 2018), memory matching (Naber et al., 2013), and stimulus brightness (Loewenfeld, 1993). A larger PD was reported during non-corresponding compared with corresponding trials, in the Simon task (i.e., D’Ascenzo et al., 2016; van Steenbergen & Band, 2013) as well as in other interference paradigms (Brown et al., 1999; Laeng et al., 2011; Siegle et al., 2004, 2008), suggesting that behavioral interference and PD share common mechanisms.

Interpretations of the spatial correspondence effect rely on the existence of a conflict between stimulus–response routes. A direct or automatic route held in long-term memory activates same-laterality responses, i.e., facilitates the activity of the effector with the same laterality as the stimulus, irrespective of task demands; at the same time, an indirect route activates responses whose laterality depends on the task-specific decisional process. In the corresponding condition, both routes activate the same response; in the non-corresponding condition, the two routes activate different responses, producing a conflict that eventually results in slower and less accurate behavioral responses (e.g., De Jong et al., 1994; for reviews, see Rubichi et al., 2006; Proctor & Vu, 2006), and in the modulation of PD (van Steenbergen & Band, 2013).

It has been repeatedly shown that interference effects can be modulated by the experimental context (e.g., Egner, 2007). Concerning the Simon effect, spatial correspondence effects in any given trial can be modulated by spatial correspondence in the previous trial, with larger interference effects when the previous trial is spatially corresponding, and no or even a reversed Simon interference when the previous trial is spatially non-corresponding (correspondence sequence effect, CSE; Gratton et al., 1992). Moreover, pupil dilation has also been observed to be modulated by CSE in the Simon paradigm, (i.e., D’Ascenzo et al., 2016, 2018; van Steenbergen & Band, 2013). It has been suggested that the detection of a conflict due to non-correspondence in the previous trial triggers a phasic arousal response, which enhances the association between stimulus features and control mechanisms, and facilitates future activation of control mechanisms (Abrahamse et al., 2016; Dignath et al., 2020; Marther & Sutherland, 2011; Verguts & Notebaert, 2009). However, a recent study that tested the association between phasic arousal and cognitive control found little evidence for the conflict-modulated learning hypothesis, and called for further studies to examine the effects of arousal on cognitive control (Brown et al., 2014).

Several studies emphasized the role of learned bindings between stimulus features, responses, and control states in the modulation of CSE (e.g., Dignath et al., 2019; Frings et al., 2020). Once created, these bindings can be retrieved and modulate performance in the following trial (Frings et al., 2020; Hommel et al., 2004). For instance, if a stimulus feature is repeated in two neighboring trials, the retrieval of the response and control state that were associated with that feature in the initial trial is facilitated; on the other hand, when changes are present from one trial to the following one, retrieval is impaired and a less pronounced modulation of interference is observed (Dignath et al., 2019; Frings et al., 2020). It is an open question whether this short term binding can be dissociated from longer term learning that follows repeated associations (Moeller & Frings, 2017). In terms of long-time learning, more pronounced modulation of interference effects has been observed when some conditions are repeated more frequently than others, or regularities are present that allow one to efficiently perform the task (e.g., the proportion of congruent trials, Logan & Zbrodoff, 1979), suggesting that stronger links have been created between perceptual, response, and monitoring processes (Frings et al., 2020).

Here, we aimed to investigate the role of stimulus novelty in the modulation of the CE. To this end, we presented pictures of animals and vehicles to participants, asking them to categorize pictures as quickly and accurately as possible, while ignoring their position. We manipulated trial-to-trial stimulus change and picture novelty, by presenting frequent and novel pictures (Fig. 1). Since previous research indicated that sequential interference was modulated by feature repetition/change (Braem et al., 2014; Dignath et al., 2019; Spapé & Hommel, 2008), we wanted to investigate whether the repetition or change in the identity of a stimulus (natural scene representing either an animal or a vehicle) in a Simon task would modulate the size of the CE in two subsequent trials. While several studies present the same stimuli across trials (e.g., shapes and colors), in real life we are exposed to a variety of objects, viewpoints, and situations, and it is a crucial task of the system to develop a mental model (Sokolov, 1963) of the environment that can be used to predict future states of the world (Friston, 2010). When the appearance of a stimulus is more frequent than that of other stimuli, the system may expect that the same stimulus would be presented in the following trials, and a violation of this prediction by a novel stimulus represents a relevant event for the system (Bradley, 2009; Friston, 2010; Sokolov, 1963). In terms of PD, the detection of novel, as compared with frequent, stimuli has been shown to elicit pupil dilation in an oddball task (Liao et al., 2016), consistently with the activation of the LC-NE system in response to stimulus novelty (Duszkiewicz et al., 2019; Vankov, 1995); on the other hand, other studies observed pupil constriction in response to novel compared with stimuli or events that had already been presented (Bradley & Lang, 2015; Ferrari et al., 2016; Gardner et al., 1974; Heaver & Hutton, 2011; Kafkas & Montaldi, 2015; Naber et al., 2013; Papesh et al., 2012; Vo et al., 2008). Here, we manipulated stimulus novelty by presenting the same scenes in the majority of trials (83% of the total trials, “frequent” condition) or showing novel scenes in a smaller number of trials (17% of the total trials, “novel” condition). The presentation of the same scenes across the majority of trials was expected to build a strong association between frequent stimuli and the associated response. When the presentation of novel stimuli calls for an evaluation, a mismatch between the (expected) frequent stimulus and the presented novel one happens, that might call for an arousal increase and activate control processes that dampen CE in the following trial, both in terms of behavioral performance and of PD (Botvinick et al., 2001; van Steenbergen & Band, 2013).

**Fig. 1:**
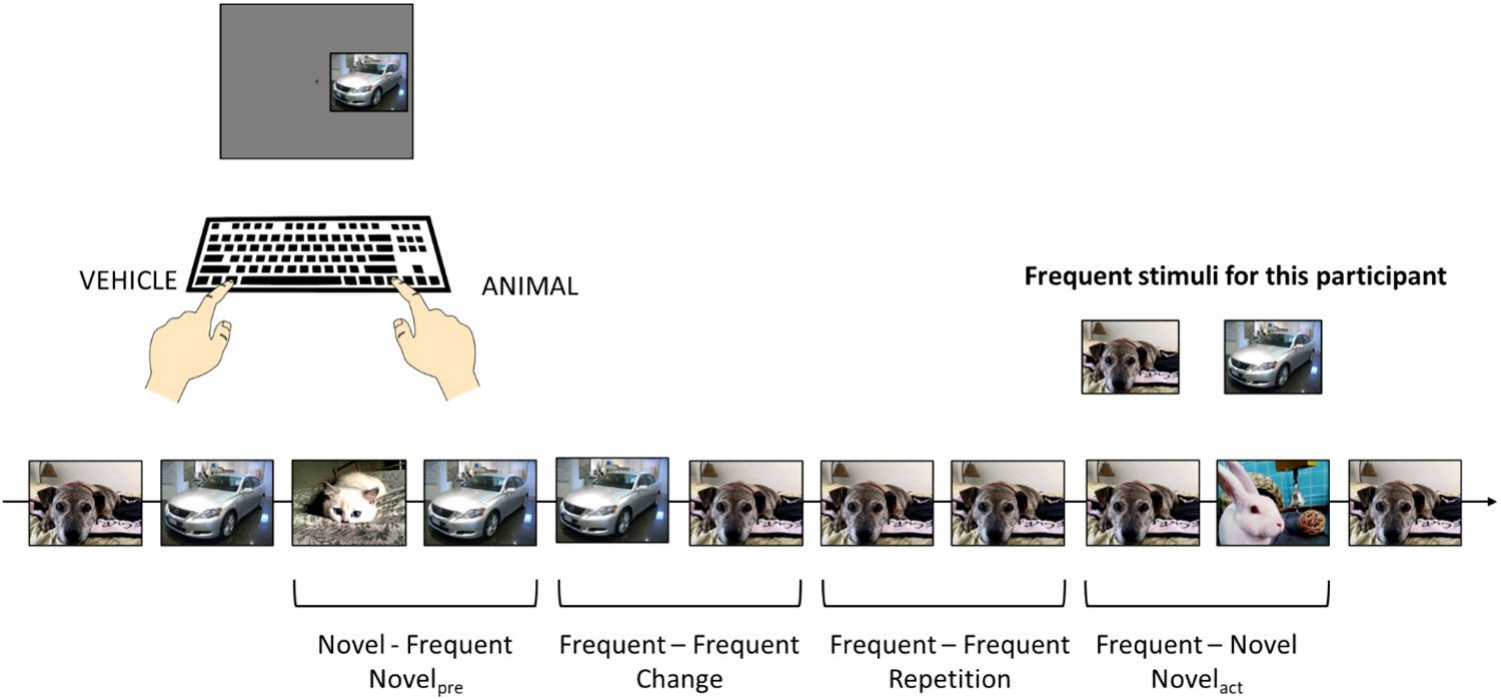
Examples of experimental conditions and associated coding. Pictures of animals or vehicles were presented on the right or left of the fixation point, and participants had to categorize them as quickly and accurately as possible using two lateralized keys on the computer keyboard (upper left). Stimulus position was task-irrelevant. Frequent and novel pictures were presented, and in the lower row the coding of sequential conditions is shown. The pictures seen as frequent stimuli by this participant are reported in the upper right, and varied in four experimental subgroups

A recent model of cognitive control builds on the creation and retrieval of bindings between stimulus features, responses, and control states (Frings et al., 2020). If this is the case, then the sequence with which stimuli are repeated or change across two neighboring trials should be crucial to the observation and modulation of interference effects. Consistently with this prediction, the repetition (or alternation) of irrelevant stimulus properties in neighboring trials has been shown to modulate interference in the following trial (Braem et al., 2014; Dignath et al., 2019; Spapé & Hommel, 2008). In a study examining the auditory Stroop, reduction of sequential interference was only observed when the gender of the task-irrelevant voice was repeated between trials, but not when it changed (Spapé & Hommel, 2008). A similar result was observed in a flanker task, in which a CSE was only observed when a task-irrelevant color surrounding the task item was repeated from one trial to the next (Braem et al., 2014). Finally, in a study in which a four-alternative flanker could be conducted either on numeric digits (i.e., 3, 4, 5, 6) or on their verbal counterparts (i.e., “three”, “four”, “five”, six”), changing the context from one trial to the next one (i.e., digit to verbal or viceversa) was associated with a less pronounced CSE compared with context repetition (same digit or verbal context in two following trials; Dignath et al., 2019). Altogether, these data support the possibility that the sequence of stimulus features (repetition/alternation) may be a major determinant of sequential interference effects, even when these features are irrelevant to the task. In the present study, we manipulated stimulus identity so that the same stimulus could be repeated in two subsequent trials, or a change could happen between the stimulus presented in the previous and the actual trial. If stimulus change plays a role, then we might expect that CSE modulation is reduced following stimulus change, as identity-laden retrieval of control states is impaired by stimulus change.

The aim of the present study was to investigate the role of stimulus novelty and of binding/retrieval mechanisms in the modulation of the CE in a Simon task, in terms of both behavioral responses and pupil dilation (PD). In the present task, to-be-categorized pictures could be animals or vehicles, and could consist in frequent stimuli (83%) or novel ones (17%). Experimental conditions, as well as predictions from a binding/retrieval and a novelty/arousal account, are depicted in Fig. 1 and reported in Table 1. In two subsequent trials, the same frequent stimulus can be presented ([Repetition]), so that there is no effect of neither stimulus change nor novelty; from both a binding/retrieval and a novelty/arousal account, this condition allows full retrieval of previous control state and does not suffer from arousal interference from novel stimuli. Starting from this baseline condition, we will address two main questions:

1. Q1. Which is the role of stimulus change in the modulation of CSE, and does it depend on stimulus novelty? In conditions in which stimulus change from the previous to the actual trial, the change can be from a frequent stimulus to a different frequent one (e.g., from the frequent car to the frequent dog in Fig. 1; [Change]) or from a novel to a frequent stimulus (e.g., from a novel cat to the frequent car in Fig. 1 [Novel_pre_]). In these conditions, the binding/retrieval account predicts that stimulus change will similarly impair the retrieval of the previous control state, leading to a reduced CSE in both conditions (Braem et al., 2014; Dignath et al., 2019; Spapé & Hommel, 2008); on the other hand, the novelty/arousal account predicts that only when the previous stimulus is novel it will activate a control mechanism that will dampen correspondence effects in the actual trial (Botvinick et al., 2001).
2. Q2. Which is the role of novelty in the modulation of correspondence effects? Here, a frequent stimulus can be followed or preceded by a novel one ([Novel_act_], [Novel_pre_]). In this condition, the predictions of a binding/retrieval account are the same as in the previous conditions, because of the change in stimulus identity; on the other hand, a novelty/arousal account would predict that the detection of a novel stimulus in the actual trial disrupts performance, leading to slower and less accurate responses, and to arousal-related pupil dilation. Moreover, to the extent to which binding and retrieval processes can be dissociated from each other (Frings et al., 2020), presenting a novel stimulus in the previous or actual trial may further impact CSE through an effect on binding (when the novel stimulus is in the previous trial) or retrieval (when the novel stimulus is in the actual trial).

**Table 1:** Experimental conditions and predictions of a change and of a novelty account

Condition	Stimulus change	Stimulus novelty	Prediction (binding/ retrieval account)	Prediction (novelty account)
Repetition	No	No	Full retrieval, full CSE	No novelty–related activation of control processes, full CSE
Change	Yes	No	Retrieval impaired by change, smaller CSE	No novelty–related activation of control processes, full CSE
Novel_pre_	Yes	Yes, in the previous trial	Retrieval impaired by change, smaller CSE; Novelty of the previous stimulus impacts binding	Novelty in the previous trial activates control processes, dampened CE in actual trial
Novel_act_	Yes	Yes, in the current trial	Retrieval impaired by change, smaller CSE; Novelty of the actual stimulus impacts retrieval	Novelty in the actual trial disrupts performance through aspecific arousal

## Experiment 1

### Method

#### Participants

A total of 30 participants (19 females, three left-handers according to the Italian version of the Edinburgh Handedness Inventory, Salmaso & Longoni, 1985) took part in the study. Age ranged from 19 to 33 (M = 21.13, SD = 2.90). All participants had normal or corrected-to-normal vision, and none of them reported current or past neurological or psychopathological problems. The participants had no previous experience with the materials used in this experiment. To determine sample size for this and the following experiments, a power analysis was conducted using G*Power (Faul et al., 2007), with the parameters alpha = 0.05, power = 0.80, partial eta squared = 0.0588 (medium effect according to Cohen, 1969), and correlation among repeated measures = 0.8, based on two independent samples of participants, for a repeated-measures ANOVA comparing the effects of Actual Trial Correspondence (corresponding, non corresponding). The power analysis indicated a minimum sample size of 15 participants. The experimental protocol conforms to the Declaration of Helsinki and was approved by the Ethical Committee of the University of Bologna.

#### Materials and design

Pictures. The stimuli were 204 pictures selected from public-domain images available on the Internet, representing outdoor animals and vehicles. Each picture was adjusted to an average brightness and contrast value (pixel intensity M = 127.5, SD = 4.51, on a 0–255 scale) and resized to 431 × 323 pixels. Of these, 4 pictures (2 animals, 2 vehicles) were used as practice trials, 8 pictures (4 animals, 4 vehicles) were to be used as frequent stimuli across trials, whereas 192 pictures (96 animals, 96 vehicles) were presented only once to each participant and used as novel stimuli. All pictures were assigned to all conditions throughout the experiment.

Stimulus repetition. Each participant performed a total of 1152 trials, of which 960 (83%) involved the presentation of the same pictures (“frequent” condition), while the rest (192 trials, 17%) presented novel images. For each participant, only 2 pictures (one animal, one vehicle) were used as frequent stimuli. Concerning frequent pictures, each participant was randomly assigned to one of four experimental groups and used two out of the eight pictures in the frequent picture set as “frequent” stimuli. A preliminary analysis failed to indicate any significant difference between the four experimental groups of participants in pupil response to frequent pictures. The number of consecutive trials with frequent stimuli varied from four to six times in a row; therefore, two consecutive trials with novel stimuli were never presented.

#### Procedure

In each trial, a picture could be presented to the left or to the right of the fixation cross. Participants were instructed to respond as quickly and as accurately as possible to the stimulus category (i.e., animal or vehicle), while ignoring their location. Responses were made by pressing the “Alt” key on a QWERTY keyboard with the index finger of their left hand or the “Control” key with the index finger of their right hand. The keyboard was located centrally in relation to the body midline. Half of the participants responded to animals with their left hand and to vehicles with their right hand, while the other half had the opposite category/response mapping.

Trial correspondence was defined based on the laterality of stimulus presentation and the sidedness of the correct response. When these were the same (e. g., picture on the right and response with the right hand), a trial was defined as “corresponding”; when they differed, e.g., when a stimulus was presented on the left and required a right-hand response, the trial was defined as “non-corresponding”. An equal number of corresponding and non-corresponding trials were presented during novel and frequent trials. Experimental sequences were built by balancing the correspondence factor in the current trial, the correspondence in the previous trial, and the novelty of the picture (novel vs. frequent). The task was preceded by 20 practice trials and was organized into 6 blocks of 192 trials each, with a short break after every block.

**Fig. 2:**
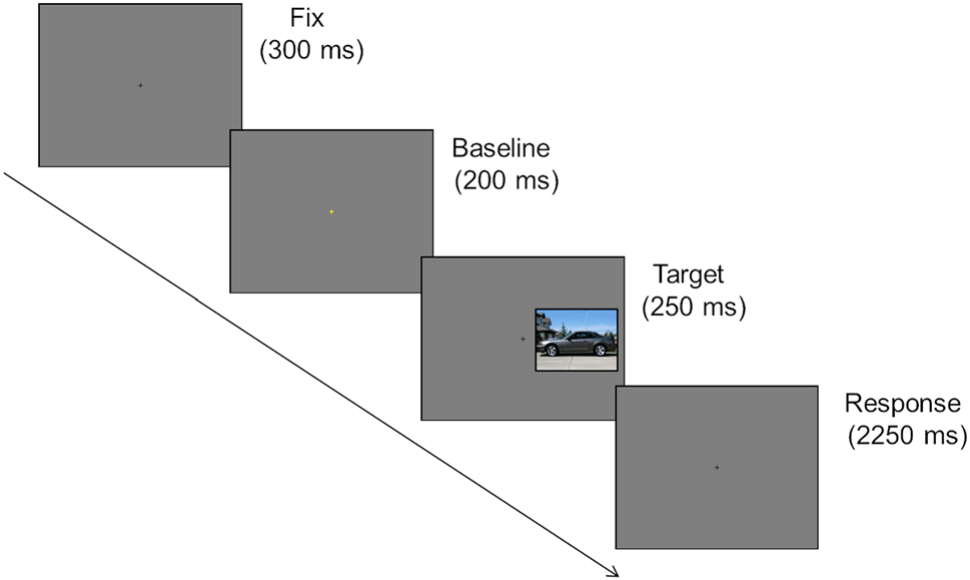
Temporal sequence of a representative trial

Each trial started with a black fixation cross at the center of the screen that, after 300 ms, turned yellow for 200 ms (warning cue) and then returned to black for the remaining duration of the trial. Then, a picture appeared for 250 ms to the left or to the right of the fixation cross. A gray blank screen was presented for 2250 ms. A trial lasted 3000 ms (see Fig. 2).

#### Apparatus

Participants were seated in front of a SMI RED 500 remote eye tracking system, positioned below a 22’’ LCD monitor (1280 × 800) which was situated approximately 60 cm from the participant’s head. The visual angle subtended by each image was 15.19 (horizontal) × 11.42 (vertical) degrees and the distance from the center (center-side) was 2 degrees. Average room illumination was 14 lx, as measured by a diode-type digital luxmeter.

#### Pupil recording and scoring

Pupil size was acquired continuously, at a rate of 250 samples per second, from the right eye. Data were converted and analysed using ILAB (Gitelman, 2002) and in-house developed Matlab routines. To identify missing or noisy data, the following procedure was used: trials with flat or missing data, or with excessive variability (exceeding ± 2.5 SD), were excluded from data analysis. Within each trial, datapoints in which blinks happened, large pupil changes (> 0.3 mm) occurred in consecutive datapoints, or where an exceedingly small pupil size was reported (< 1 mm), were excluded and replaced by linear interpolation starting from 5 samples before and ending 5 samples after (van Orden et al., 2000). If any trial contained more than 50% interpolated data, then the whole trial was discarded from data analysis. Overall, these criteria led to the removal of 3.7% of total trials.

The baseline pupil size was defined as the average pupil size (mm) in the 500 ms interval preceding stimulus onset (time 0). In each trial, the baseline pupil size was linearly subtracted from the pupil diameter changes following stimulus onset. Picture presentation elicited pupil constriction (light reflex), which was descriptively maximal around 810 ms (latency based on the grand-averaged pupil waveform across conditions; collapsed localizer approach, Luck & Gaspelin, 2017) and then returned to baseline, reaching a plateau around 1400 ms after stimulus onset. Based on these latencies, we examined the time intervals 750–900 ms (peak of light reflex), 900–1400 (recovery from light reflex), 1400–2500 (plateau).

#### Statistical analysis

Practice trials, first trial of each block, errors, trials following an error, and discarded pupil trials were excluded from the analysis. Response times which were 2.5 SD faster or slower than the participant’s mean were excluded from the analysis of response times. The average number of discarded trials per participant was 145 (out of 1152). Five participants that had 80% or less retained data were discarded from the analysis.

For all analyses, repeated-measures ANOVAs were carried out on pupil dilation (PD), response times (RTs), and accuracy, with Huynh–Feldt correction when appropriate. The partial eta squared statistic (*η*^2^_p_), indicating the proportion between the variance explained by one experimental factor and the total variance, was calculated and reported.

Additionally, we quantified the Bayes factor (BF) using JASP 0.16.4 (JASP Team, 2018). For each p value resulting from ANOVAs, we report the corresponding Bayes factor for exclusion (BF_excl_) comparing models include the term of interest against models that do not include the term of interest, and excluding higher-order interactions (Mathôt, 2017; van den Bergh et al., 2020).

Data analysis followed the design described in Fig. 1 and in Table 1, and examined the effects of Previous Trial Correspondence (corresponding [C_pre_] vs. non corresponding [NC_pre_]), Actual Trial Correspondence (corresponding [C_act_] vs. non corresponding [NC_act_]), and Repetition Condition (frequent, same picture in the previous and in the actual trial [Repetition]; frequent, but different pictures in the previous and in the actual trial [Change]; frequent picture preceded by a novel picture [Novel_pre_]; novel picture in the actual trial [Novel_act_]). If a superordinate main effect or interaction was significant, we proceeded to ANOVAs on subordinate conditions or to post-hoc comparisons. Pairwise comparisons, including post-hoc calculation of the Simon effect as the difference in response times between corresponding and non-corresponding conditions, were computed through paired t-tests.

### Results of Experiment 1

#### Response times

**Fig. 3:**
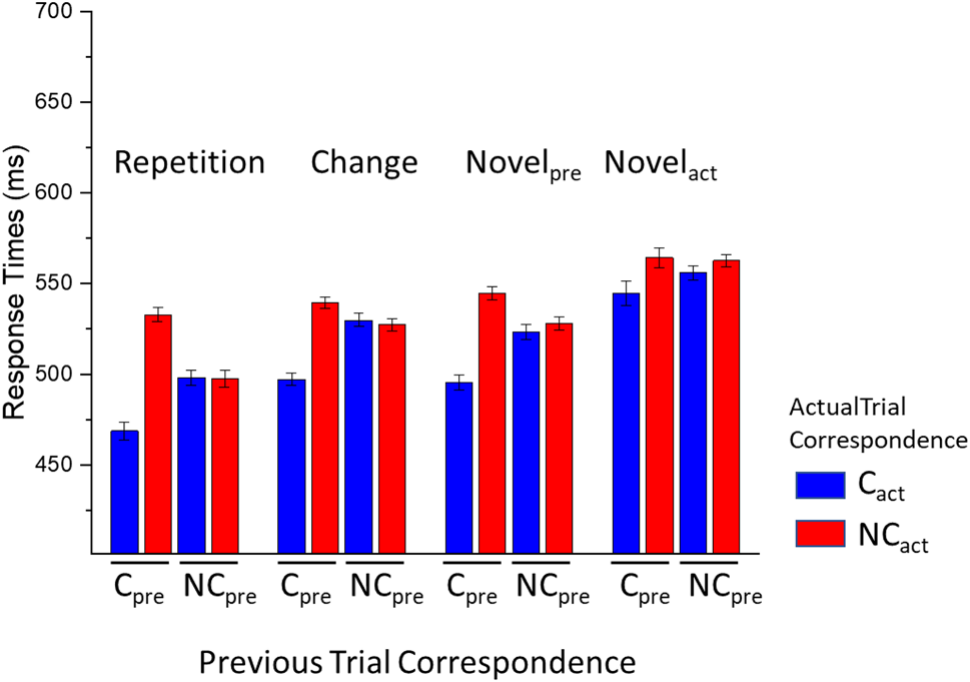
Response times in Experiment 1. Error bars represent within-participants standard error of the mean (O’Brien & Cousineau, 2014)

The results for Response Times are reported in Fig. 3. A significant effect of Actual Trial Correspondence was observed, F(1, 24) = 36.01, p < 0.001, *η*^*2*^_*p*_ = 0.6, BF_excl_ < 0.001, with slower responses for non corresponding compared with corresponding trials. No significant effect of Previous Trial Correspondence was observed, F(1, 24) = 2.6, p = 0.12, *η*^*2*^_*p*_ = 0.1, BF_excl_ = 2.791. A significant interaction between Actual and Previous Trial Correspondence was observed, F(1, 24) = 77.1, p < 0.001, *η*^*2*^_*p*_ = 0.76, BF_excl_ < 0.001, with significant differences between corresponding and non corresponding trials when the previous trial was corresponding, t(24) = 8.53, p < 0.001, but no significant difference between corresponding and non corresponding trials when the previous trial was non corresponding, t(24) = 0.169, p = 0.868.

A significant effect of Repetition Condition was observed, F(3, 72) = 76.85, p < 0.001, *η*^*2*^_*p*_ = 0.76, BF_excl_ < 0.001, indicating significantly slower responses for Novel_act_ trials compared with all other conditions, ts(24) > 6.26, p < 0.001, and fastest responses to Repetition trials compared with all other conditions, ts(24) < -8.19, ps < 0.001. Change and Novel_pre_ trials were intermediate in latency, and did not differ from each other, t(24) = -0.61, p = 0.548. We also observed significant interactions between Repetition Condition and Previous Trial Correspondence, F(3, 72) = 4.24, p = 0.008, *η*^*2*^_*p*_ = 0.15, BF_excl_ = 1.415, indicating that in Change trials slower responses were observed following NC_pre_ compared with C_pre_ trials, F(1, 24) = 18.433, p < 0.001, *η*^*2*^_*p*_ = 0.434, BF_excl_ = 0.225, while no significant effect of Previous Trial correspondence was observed in all other Repetition Conditions, Fs(1, 24) < 0.18, ps > 0.675, *η*^*2*^_*p*_s < 0.007, BF_excl_s > 3.428. A significant interaction between Repetition Condition and Actual Trial Correspondence was also observed, F(3, 72) = 5.57, p = 0.01, *η*^*2*^_*p*_ = 0.19, BF_excl_ = 0.058, indicating significantly slower responses for corresponding compared with non corresponding trials in all conditions, ts(24) > 4.62, ps < 0.001, but not in Novel_act_ trials, ts(24) = 1.41, p = 0.169.

A significant three-way interaction between Repetition Condition, Previous Trial Correspondence and Actual Trial Correspondence was observed, F(3, 72) = 18.92, p < 0.001, *η*^*2*^_*p*_ = 0.44, BF_excl_ < 0.001. Focusing on conditions preceded by corresponding trials, a significant interaction between Repetition Condition and Actual Trial Correspondence was observed, F(3, 72) = 20.467, p < 0.001, *η*^*2*^_*p*_ = 0.460, BF_excl_ < 0.001. Significant differences between corresponding and non corresponding trials were observed in all conditions, ts(24) > 7.385, ps < 0.001, except in the Novel_act_ condition, t = 1.761, p = 0.091. Pairwise comparing the effects of Actual Trial Correspondence between each couple of Repetition Conditions, significant interactions between Repetition Condition and Actual Trial Correspondence were observed when comparing Repetition and Change trials, F(1, 24) = 8.889, p = 0.006, *η*^*2*^_*p*_ = 0.270, BF_excl_ = 0.048; Repetition and Novel_pre_, F(1, 24) = 5.757, p = 0.025, *η*^*2*^_*p*_ = 0.193, BF_excl_ = 0.277; Repetition and Novel_act_, F(1, 24) = 39.475, p < 0.001, *η*^*2*^_*p*_ = 0.622, BF_excl_ < 0.001; Change and Novel_act_, F(1, 24) = 24.666, p < 0.001, *η*^*2*^_*p*_ = 0.507, BF_excl_ < 0.001; Novel_pre_ and Novel_act_, F(1, 24) = 34.545, p < 0.001, *η*^*2*^_*p*_ = 0.590, BF_excl_ < 0.001; no significant difference was observed between Change and Novel_pre_, F(1, 24) = 1.542, p = 0.226, *η*^*2*^_*p*_ = 0.060, BF_excl_ = 1.859. Significant interactions between Previous Trial Correspondence and Actual Trial Correspondence were observed in all repetition conditions, F(1, 24) > 21.41, ps < 0.001, *η*^*2*^_*p*_ > 0.47, BF_excl_s < 0.001 except Novel_act_, F(1, 24) = 2.2, p = 0.151, *η*^*2*^_*p*_ = 0.08, BF_excl_ = 1.388. In conditions preceded by non corresponding trials, no significant interaction between Repetition Condition and Actual Trial Correspondence was observed, F(3, 72) = 1.485, p = 0.235, *η*^*2*^_*p*_ = 0.058, BF_excl_ = 3.123.

#### Error rate

Error rates are reported in Table 2. A significant effect of Actual Trial Correspondence was observed, F(1, 24) = 21.88, p < 0.001, *η*^*2*^_*p*_ = 0.4, BF_excl_ = 0.003, indicating less accurate responses for non corresponding compared with corresponding trials. A significant effect of Previous Trial Correspondence indicated more accurate responses after a non corresponding trial than after a corresponding trial, F(1, 24) = 5.74, p = 0.025, *η*^*2*^_*p*_ = 0.19, BF_excl_ = 0.971. Finally, a significant two-way interaction between Previous and Actual Trial Correspondence was observed, F(1, 24) = 41.7, p < 0.001, *η*^*2*^_*p*_ = 0.63, BF_excl_ < 0.001, indicating significant effects of Actual Trial Correspondence after a corresponding trial, F(1, 24) = 48.68, p < 0.001, *η*^*2*^_*p*_ = 0.67, BF_excl_ < 0.001, but not after a non corresponding trial, F(1, 24) = 0.288, p = 0.597, *η*^*2*^_*p*_ = 0.012, BF_excl_ = 3.498.

**Table 2:** Error rates (mean and standard deviation) for Experiment 1

				Actual trial correspondence			
		Corresponding [C_act_]		Non corresponding [C_act_]		Total	
Repetition condition	Previous trial correspondence	M	SD	M	SD	M	SD
Repetition	Corresponding [C_pre_]	1.14	1.90	5.99	4.35	3.57	2.70
	Non Corresponding [NC_pre_]	2.79	2.55	2.13	1.75	2.46	1.60
Change	Corresponding [C_pre_]	1.17	1.50	5.7	4.05	3.43	2.35
	Non Corresponding [NC_pre_]	3.46	3.30	3.26	2.75	3.36	2.65
Previous Novel [Novel_pre_]	Corresponding [C_pre_]	1.56	1.85	7.56	4.90	4.56	2.60
	Non Corresponding [NC_pre_]	3.11	3.50	3.34	2.85	3.22	2.45
Actual Novel [Novel_act_]	Corresponding [C_pre_]	2.17	3.30	4.04	4.00	3.11	2.95
	Non Corresponding [NC_pre_]	3.11	4.00	2.69	3.60	2.9	3.10
Total		2.31	2.00	4.34	2.45		

No significant effect of Repetition Condition was observed, F(3, 72) = 2.39, p = 0.096, *η*^*2*^_*p*_ = 0.09, BF_excl_ = 2.754. A significant three-way interaction between Repetition Condition, Previous Trial Correspondence and Actual Trial Correspondence was observed, F(3, 72) = 2.81, p = 0.047, *η*^*2*^_*p*_ = 0.1, BF_excl_ = 0.262. In conditions preceded by corresponding trials, a significant interaction between Repetition Condition and Actual Trial Correspondence was observed, F(1, 24) = 5.564, p = 0.002, *η*^*2*^_*p*_ = 0.191, BF_excl_ = 0.014. In conditions preceded by non corresponding trials, no interaction of Repetition Condition and Actual Trial Correspondence was observed, F(1, 24) = 0.346, p = 0.772, *η*^*2*^_*p*_ = 0.014, BF_excl_ = 11.82. Focusing on each repetition condition, significant interactions of Previous and Actual Trial Correspondence were observed in all conditions, Fs(1, 24) > 16.91, ps < 0.001, *η*^*2*^_*p*_s > 0.41, BF_excl_s < 0.001 except for the Novel_pre_ condition, F(1, 24) = 4.07, p = 0.055, *η*^*2*^_*p*_ = 0.15, BF_excl_ = 0.408. In the Change, Repetition, and Novel_pre_ condition less accurate performance was observed for non corresponding compared with corresponding trials when these were preceded by corresponding trials, ts > 5.686, ps < 0.001, but not when they were preceded by non corresponding trials, ts < 0.269, ps > 0.277.

A significant interaction was observed between Repetition Condition and Previous Trial Condition, F(3, 72) = 2.03, p = 0.118, *η*^*2*^_*p*_ = 0.08, BF_excl_ = 3.294, with significantly less accurate responses following corresponding than non corresponding trials in the Novel_pre_ and Repetition conditions, Previous Trial Condition Fs(1, 24) > 5.96, ps < 0.022, *η*^*2*^_*p*_s > 0.2, BF_excl_s > 1.064, but no significant effect of Previous Trial Condition in the Change and Novel_act_ conditions, Fs(1, 24) < 0.13, ps > 0.722, *η*^*2*^_*p*_s < 0.01, BF_excl_s > 3.723. Finally, a significant interaction was observed between Repetition Condition and Actual Trial Condition, F(3, 72) = 3.86, p = 0.02, *η*^*2*^_*p*_ = 0.14, BF_excl_ = 0.399, indicating significantly less accurate responses for non corresponding compared with corresponding trials in all conditions, Fs(1, 24) > 13.92, ps < 0.001, *η*^*2*^_*p*_s > 0.37, BF_excl_s < 0.082, except in the Novel_act_ condition, F(1,24) = 1.14, p = 0.297, *η*^*2*^_*p*_ = 0.05, BF_excl_ = 2.370.

#### Pupil dilation

**Fig. 4:**
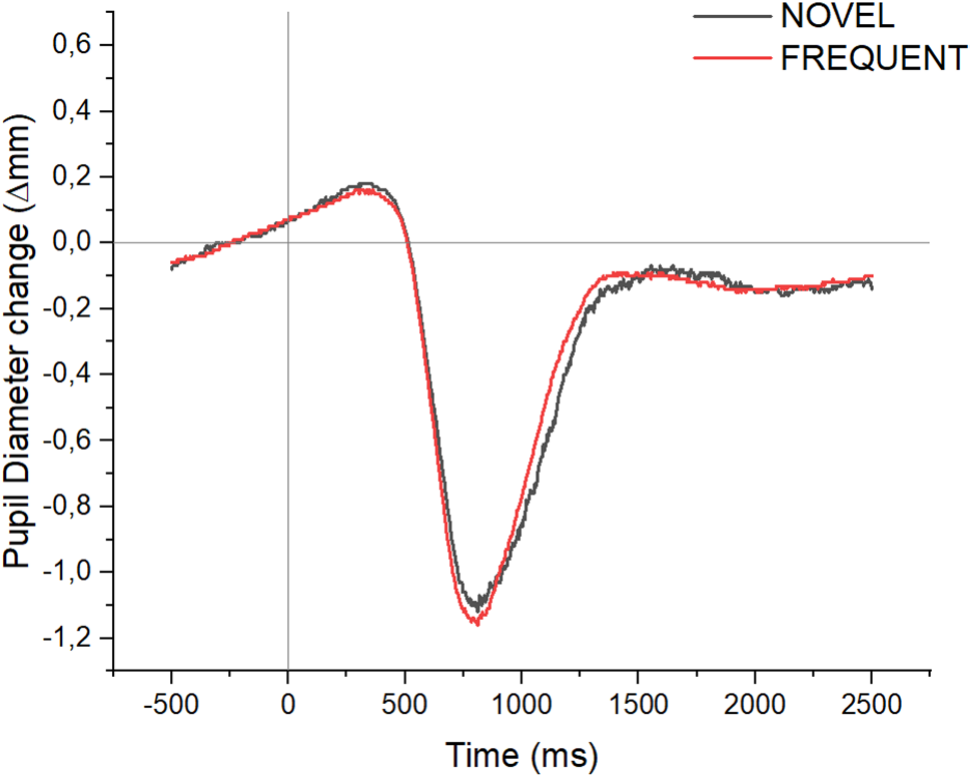
Pupil diameter change for novel and frequent trials in Experiment 1

Waveforms for pupil size modulation by novelty are reported in Fig. 4, and means and SDs for each condition and time of interest are reported in Supplementary Table 1. In the light reflex interval (750–900 ms), no significant main effect or interaction was observed, Fs < 1.912, ps > 0.179, *η*^*2*^_*p*_s < 0.074, BF_excl_s > 1.976. In the time interval from 900 to 1400 ms, a significant main effect was observed for Repetition Condition, F(3, 72) = 3.873, p = 0.027, *η*^*2*^_*p*_ = 0.139, BF_excl_ = 0.360, with significantly more constricted pupil diameter in the Novel_act_ condition compared with both the Change and Repetition conditions, ts(24) > 2.15, ps < 0.042, and no difference between Novel_act_ and Novel_pre_, nor among all other pairs of conditions, ts(24) < 1.75, p > 0.092; no other main effect or interaction was significant in the 900–1400 ms time interval, Fs < 2.128, ps > 0.104, *η*^*2*^_*p*_s < 0.081, BF_excl_s > 2.173. Finally, in the 1400–2500 time interval, no significant main effect or interaction was observed, Fs < 2.614, ps > 0.061, *η*^*2*^_*p*_s < 0.098, BF_excl_s > 0.674.

### Discussion

In Experiment 1, we investigated the effects of stimulus change and novelty on the sequential modulation of the Simon effect. Stimulus change could be due to a change from a novel to a frequent stimulus, or from one frequent stimulus to the other frequent stimulus (Fig. 1); in both cases, we observed a reduced correspondence sequential effect compared with trials which presented the same frequent stimulus that was shown in the previous trial. These data are consistent with previous studies, which observed a reduction in CSE in trials in which a change in context happened compared with trials in which the same context was repeated over trials (Dignath et al., 2019; Spapé & Hommel, 2008). Here, the reduction in CSE was due to the reduced CE following corresponding trials in changing contexts (Change and Novel_pre_, compared with Repetition). Moreover, the reduction in CE and CSE in trial preceded by either novel or different frequent stimuli did not differ from each other, suggesting that the added relevance of stimulus novelty did not affect the CE dampening which was due to context change.

The presentation of novel stimuli in the actual trial determined slower responses which were not modulated by spatial correspondence, and reduced correspondence effects in the following trial. Moreover, a moderate effect of repetition condition was observed on PD, however with more pronounced constriction for novel compared with frequent pictures (Ferrari et al., 2016), suggesting a role of memory processes rather than arousal in this particular paradigm; however, this effect was only evident when pupil constriction was recovering from the LR, and likely reflected a slightly different recovery time for novel as compared with frequent stimuli. All in all, the results of Experiment 1 indicate that while stimulus change determined a modulation in the correspondence effect in the subsequent trial, we observed no clear evidence indicating that novelty-related arousal further modulated CSE.

In Experiments 2 and 3, we increased the arousing value of novel stimuli, by associating a change in required response for frequent and novel stimuli; in particular, frequent pictures required participants to respond using the same manual modality as in Experiment 1, while novel pictures required participants to vocally respond to the category of the visual stimulus. All novel stimuli were thus associated with a prediction violation at the level of response effector selection (from the more frequent manual modality to the less frequent vocal modality). Novel trials were therefore lacking a manual response (as in Hommel 2004, exp. 3), and required participants to switch from a manual response modality to a vocal response modality (Braem et al., 2011).

It was expected that the change in response modality determined a stronger violation of experimental sequence of events, therefore determining a higher arousal level which might reflect on pupil dilation. With a stronger manipulation of arousal, we expected that if arousal further modulates CE in addition to stimulus change, then a stronger dampening of CE should be observed following novel (vocal response) trials. On the other hand, if modulation of CE is associated with change, then results similar to Experiment 1 should be observed.

## Experiment 2

### Method

#### Participants

A total of 22 participants (16 females, two left handers) took part in the study. Age ranged from 20 to 26 (M = 21.18, SD = 1.56). All participants had normal or corrected-to-normal vision, and none of them reported current or past neurological or psychopathological problems. The participants had no previous experience with the materials used in this experiment. The experimental protocol conforms to the Declaration of Helsinki and was approved by the Ethical Committee of the University of Bologna.

#### Materials and design

Pictures and Stimulus novelty were the same as those used in Experiment 1.

#### Procedure

Participants were instructed to respond as quickly and as accurately as possible to the stimulus category (i.e., animal or vehicle), while ignoring its location. For frequent stimuli, as in Experiment 1, responses were made by pressing the “Alt” key on a QWERTY keyboard with the index finger of their left hand or the “Control” key with the index finger of their right hand. Half of the participants responded to animals with their left hand and to vehicles with their right hand, while the other half had the opposite mapping. For novel stimuli, responses were made using a vocal response, by saying ‘animal’ or ‘vehicle’. Vocal responses were recorded but are not analyzed in the present paper.

### Apparatus and Pupil recording and scoring

See Experiment 1.

#### Statistical analysis

Practice trials, first trial of each block, errors, trials following an error, and discarded pupil trials were excluded from the analysis. Response times which were more than 2.5 SD faster or slower than the participant’s mean were excluded from the analysis of response times. The average number of discarded trials per participant was 109 (out of 1152). Three participants that had 80% or less retained data were discarded from the analysis.

Data analysis for behavioral responses (response times and error rate) was organized as in Experiment 1, with the exception of the Novel_act_ conditions which did not require a manual response and therefore were not included in the data analysis design for response times. Therefore, the full-factorial design of Experiment 1 was analyzed using the condition listed in Table 3. For response times and accuracy, a factor Repetition Condition with five levels (same frequent picture in previous and actual trial with previous trial being a corresponding one [Repetition-C_pre_]; same frequent picture in previous and actual trial with previous trial being a non corresponding one [Repetition-NC_pre_]; different frequent pictures in previous and actual trial, with previous trial being a corresponding one [Change-C_pre_]; different frequent pictures in previous and actual trial, with previous trial being a non corresponding one [Change-NC_pre_]; frequent picture preceded by a novel picture [Novel_pre_]), and a factor Actual Trial Correspondence (corresponding [C_act_]; non corresponding [NC_act_]).

**Table 3:** Analytical design for Experiments 2 and 3

		Preceding trial		Actual trial		
Experiment	Repetition condition	Novelty	Correspondence	Novelty	Correspondence	Dependent Variable
Exp 2 & 3	Novel_pre_	novel	–	frequent	corresponding	RTs, error rate, PD
	Novel_pre_	novel	–	frequent	non corresponding	RTs, error rate, PD
	Change	frequent	corresponding	frequent	corresponding	RTs, error rate, PD
	Change	frequent	corresponding	frequent	non corresponding	RTs, error rate, PD
	Change	frequent	non corresponding	frequent	corresponding	RTs, error rate, PD
	Change	frequent	non corresponding	frequent	non corresponding	RTs, error rate, PD
	Repetition	frequent	corresponding	frequent	corresponding	RTs, error rate, PD
	Repetition	frequent	corresponding	frequent	non corresponding	RTs, error rate, PD
	Repetition	frequent	non corresponding	frequent	corresponding	RTs, error rate, PD
	Repetition	frequent	non corresponding	frequent	non corresponding	RTs, error rate, PD
	Novel_act_	frequent	corresponding	novel	–	PD only
	Novel_act_	frequent	non corresponding	novel	–	PD only
Exp 3 only	Novel_both_	novel	–	novel	–	PD only

Concerning pupil data, the conditions in which a behavioral response was not collected but pupil data were available were added to the analytical design, and the design therefore included an overarching Condition factor including the levels listed in Table 3. Only if the main effect of this overarching factor was significant, we proceeded to analyze a more restricted set of conditions using post-hoc t-tests.

### Results of Experiment 2

#### Response times

**Fig. 5:**
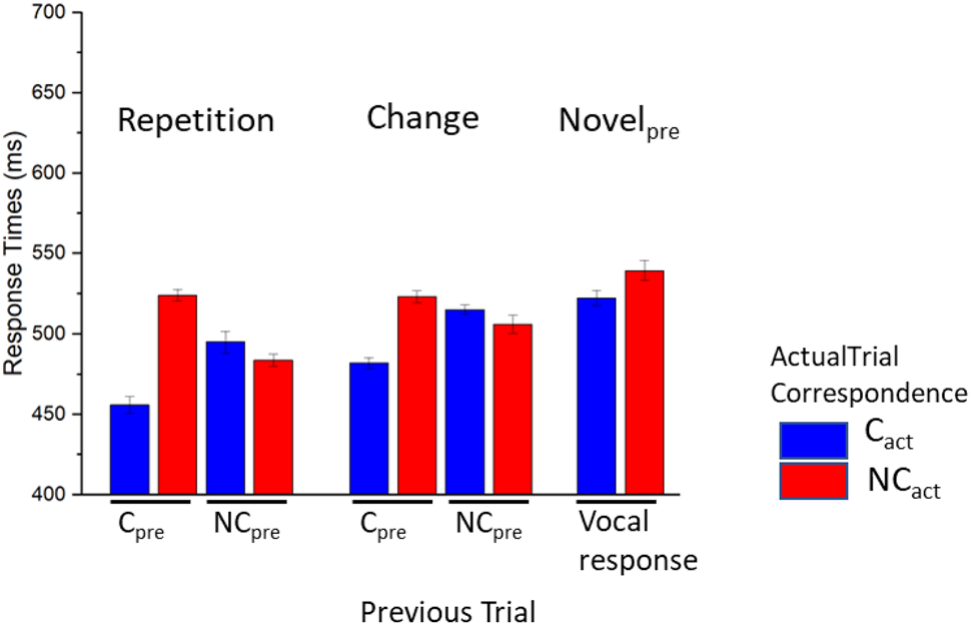
Response Times in Experiment 2. Error bars represent within-participants standard error of the mean

Response Times are reported in Fig. 5. A significant effect of Actual Trial Correspondence was observed, with slower responses after non corresponding compared with corresponding trials, F(1, 18) = 22.21, p < 0.001, *η*^*2*^_*p*_ = 0.55, BF_excl_ = 0.015. A significant main effect of Repetition Condition was observed, F(4, 72) = 23.20, p < 0.001, *η*^*2*^_*p*_ = 0.56, BF_excl_ < 0.001, with slower responses to Novel_pre_ trials compared with all other conditions, ts(18) > 3.47, ps < 0.003, to trials in both Change-C_pre_ and Change-NC_pre_ conditions compared with both Repetition-C_pre_ and Repetition-NC_pre_ conditions, ts(18) > 3.43, ps < 0.003, and to trials in the Change-NC_pre_ compared with Change-C_pre_ trials, t(18) = 2.83, p = 0.011.

A significant interaction between Repetition Condition and Actual Trial Correspondence was observed, F(4, 72) = 47.14, p < 0.001, *η*^*2*^_*p*_ = 0.72, BF_excl_ < 0.001. Following this significant interaction, slower responses were observed for non corresponding compared with corresponding trials in each repetition condition, yielding significant differences in Repetition-C_pre_, Change-C_pre_ and Novel_pre_ conditions, ts(18) > 2.555, ps < 0.02, but not in the Repetition-NC_pre_ and Change-NC_pre_ conditions, ts < -1.689, ps > 0.108. Moreover, pairwise comparing the correspondence effect between each combination of repetition conditions, no difference was observed between the two conditions preceded by non corresponding trials, Repetition Condition x Actual Trial Correspondence F(1, 18) = 0.14, p = 0.709, *η*^*2*^_*p*_ = 0.008, BF_excl_ = 2.871. Comparing Repetition-C_pre_, Change-C_pre_, and Novel_pre_ conditions, we observed significant differences between each of these conditions and the two conditions preceded by non corresponding trials, Repetition Condition x Actual Trial Correspondence Fs(1, 18) > 10.82, ps < 0.004, *η*^*2*^_*p*_s > 0.38, BF_excl_s < 0.037, and among each combination of these conditions, Repetition Condition x Actual Trial Correspondence Fs(1, 18) > 29.13, ps < 0.001, *η*^*2*^_*p*_s > 0.62, BF_excl_s < 0.006, with most pronounced effects of correspondence for Repetition-C_pre_ trials (M = 69.95, SD = 31.11), intermediate for Change-C_pre_ trials (M = 40.99, SD = 23.11) and least pronounced for Novel_pre_ trials (M = 16.99, SD = 28.98).

#### Error rate

Error rates for all conditions are reported in Table 4. A significant effect of Actual Trial Correspondence was observed, F(1, 18) = 20.57, p < 0.001, *η*^*2*^_*p*_ = 0.53, BF_excl_ = 0.021, with a higher error rate after non corresponding compared with corresponding trials. A significant main effect of Repetition Condition was observed, F(4, 72) = 6.86, p < 0.001, *η*^*2*^_*p*_ = 0.28, BF_excl_ = 0.032. All differences between conditions were significant, ts(18) > 2.95, ps < 0.008, except for the difference between Novel_pre_ and Repetition-C_pre_, t(18) = 0.64, p = 0.53, Change-C_pre_ and Change-NC_pre_, t(18) = 0.32, p = 0.75, Change-NC_pre_ and Repetition-NC_pre_, t(18) = -0.40, p = 0.695, and Change-C_pre_ compared with Repetition-NC_pre_, t(18) = 0.09, p = 0.926.

**Table 4:** Error rates for Experiment 2

				Actual trial correspondence			
		Corresponding [C_act_]		Non corresponding [NC_act_]		Total	
Repetition condition	Previous trial correspondence	M	SD	M	SD	M	SD
Repetition	Corresponding [C_pre_]	1.095	1.560	6.672	3.808	3.884	2.431
	Non Corresponding [NC_pre_]	2.405	2.242	1.760	1.396	2.082	1.476
Change	Corresponding [C_pre_]	0.867	1.444	3.381	2.538	2.124	1.718
	Non Corresponding [NC_pre_]	2.360	1.790	2.142	1.987	2.251	1.489
Novel [Novel_pre_]	–	2.815	2.142	4.228	2.806	3.522	1.973
Total		1.908	1.209	3.637	1.869		

A significant interaction between Repetition Condition and Actual Trial Correspondence was observed, F(4, 72) = 21.83, p < 0.001, *η*^*2*^_*p*_ = 0.55, BF_excl_ < 0.001. Following this significant interaction, a higher error rate was observed for non corresponding trials compared with corresponding ones in Change-C_pre_ and Repetition-C_pre_ conditions, ts(18) > 4.78, ps < 0.001, but not in the Change-NC_pre_ and Repetition-NC_pre_ conditions, ts(18) < 1.228, p > 0.235, nor in the Novel_pre_ condition, t(18) = 2.02, p = 0.059. Moreover, pairwise comparing the correspondence effect among all repetition conditions, no difference was observed between the two conditions preceded by non corresponding trials, Repetition Condition x Actual Trial Correspondence F(1, 19) = 0.334, p = 0.570, *η*^*2*^_*p*_ = 0.017, BF_excl_ = 2.693. We observed significant differences between both the Repetition-NC_pre_ and the Change-NC_pre_ condition when compared against each of the remaining three conditions (Repetition-C_pre_, Change-C_pre_, and Novel_pre_) Repetition Condition x Actual Trial Correspondence Fs(1, 18) > 8.269, ps < 0.01 *η*^*2*^_*p*_s > 0.32, BF_excl_s < 0.114. Finally, significant differences in the magnitude of the correspondence effect were observed between the Change-C_pre_ and the Repetition-C_pre_ condition, F(1, 18) = 30.773, p < 0.001, *η*^*2*^_*p*_ = 0.631, BF_excl_ = 0.002, between Repetition-C_pre_ and Novel_pre_ trials, F(1, 18) = 20.24, p < 0.001, *η*^*2*^_*p*_ = 0.53, BF_excl_ < 0.001, but not between Change-C_pre_ and Novel_pre_ trials, F(1, 18) = 1.834, p = 0.192, *η*^*2*^_*p*_ = 0.09, BF_excl_ = 1.202.

#### Pupil dilation

**Fig. 6:**
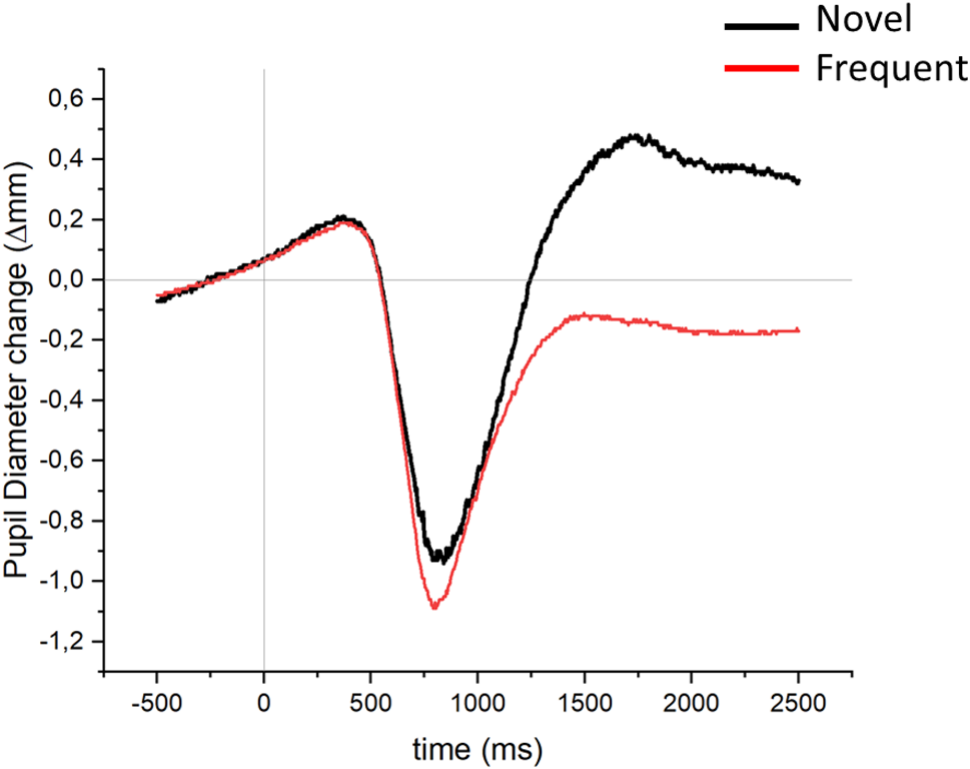
Pupil diameter change for novel and frequent trials in Experiment 2

Waveforms for the pupil modulation by actual trial novelty are reported in Fig. 6, and means and SDs for each condition and time interval are reported in Supplementary Table 2. For the time interval 750–900, the effect of Condition was not significant, F(11, 198) = 2.055, p = 0.062, *η*^*2*^_*p*_ = 0.102, BF_excl_ = 0.999. Significant effects of Condition were observed in the 900–1400 time interval, F(11, 198) = 3.579, p = 0.026, *η*^*2*^_*p*_ = 0.166, BF_excl_ = 0.007, and especially in the 1400–2500 time interval, F(11, 198) = 14.976, p < 0.001, *η*^*2*^_*p*_ = 0.454, BF_excl_ < 0.001, mostly reflecting a more pronounced pupil dilation for novel as compared with frequent trials. Post hocs for this effect are reported in Supplementary Table 2.

### Discussion

In Experiment 2, we introduced a stronger violation in experimental sequence of events by instructing participants to change from a manual effector used in 83% of the total trials (frequent pictures), to a vocal effector for the remaining 17% trials (novel pictures), while keeping all other details identical to Experiment 1. The change in response effector in rare novel trials was associated with a strong increase in pupil dilation compared with frequent trials, possibly related to the higher arousing value, or with the motor demands associated with the rare vocal response. In terms of behavioral responses, a dampening in the size of the Simon effect was observed in trials following vocal responses, although spatial correspondence effects on response times were still significant (Hommel, 2004, exp. 3). Importantly, the dampening in correspondence effect following novel trials was more pronounced than following change trials with frequent stimuli.

The results of Experiment 2 suggest that the Simon effect can be modulated by a rare effector change (vocal response to novel scenes) in the preceding trial. However, different theoretical accounts may explain this result. First, the stronger violation which was introduced by effector change may have induced a more pronounced arousal state (possibly reflected in pupil modulation) that dampened correspondence effect in the following trial. However, omitting the response is an experimental manipulation akin to task-switching, and it has been suggested that competing motor demands in subsequent trials may reduce cognitive control, therefore determining stronger effects compared with when only stimulus features change between neighboring trials (Frings et al., 2020). Finally, it is an open question whether the type of inter-trial learning that modulates correspondence effects can be modulated by a long-term, more sustained learning that is sensitive to factors such as relative stimulus probabilities.

To disentangle these three possibilities, in Experiment 3 we replicated the design of Experiment 2, modifying the relative probability of frequent pictures (manual response) and novel pictures (vocal response), which were both set at 50% probability. If the reduction in correspondence effects in the actual trial which was observed in Experiment 2 is due to increased arousal in novel (vocal) trials, then the reduction in the novelty of vocal trials should reduce arousal level, leading to a correspondence modulation similar to Experiment 1. On the other hand, if the reduction in correspondence effects in the actual trial is due to the short-term binding between motor demands in the actual and previous trial, then no difference should be observed between Experiment 2 and 3, as only short-term bindings between close trials should modulate correspondence effects. Finally, if longer-term learning modulates correspondence effects, then it can be expected that the stronger association of novel trials to vocal responses (which is achieved through practice) might determine stronger dampening of correspondence effects in Experiment 3 compared with Experiment 2. As PD might also be modulated by either arousal or motor demands, Experiment 3 will also disentangle the role of these two factors in the observed modulation of PD.

## Experiment 3

### Method

#### Participants

A total of 20 participants (14 females, all right handers) took part in the study. Age ranged from 20 to 26 (M = 21.55, SD = 1.85). All participants had normal or corrected-to-normal vision, and none of them reported current or past neurological or psychopathological problems. The participants had no previous experience with the materials used in this experiment. The experimental protocol conforms to the Declaration of Helsinki and was approved by the Ethical Committee of the University of Bologna.

#### Materials and design

Pictures. The stimuli were 596 pictures, which included the stimuli used in Experiments 1 and 2, with the addition of 392 new pictures. New stimuli were selected from public-domain images available on the Internet, representing outdoor and indoor animals and vehicles. Each picture was adjusted to an average brightness and contrast value (pixel intensity M = 127.5, SD = 4.51, on a 0–255 scale) and resized to 431 × 323 pixels. Of these, 12 pictures (6 animal, 6 vehicles; 10 were used as novel stimuli and 2 as frequent stimuli) were used as practice trials, 8 pictures (4 animal, 4 vehicle) were to be used as frequent stimuli across trials, whereas 576 pictures (288 animal, 288 vehicles) were presented only once and used as novel stimuli.

Stimulus Novelty. Each participant performed a total of 1152 trials, of which half (576, 50%) involved the presentation of frequent pictures, while the other half (576, 50%) presented novel images. For each participant, only 2 pictures (one animal, one vehicle) were used as frequent stimuli. Throughout the experimental sample, each of the 8 pictures in the frequent set was used.

#### Procedure

Except for stimulus probability, the procedure was identical to that used in Experiment 2. As the amount of novel trials increased compared with Experiment 2, it was now allowed that two novel stimuli were repeated in a row.

#### Apparatus and pupil recording and scoring

See Experiments 1 and 2.

#### Statistical analysis

Practice trials, first trial of each block, errors, trials following an error, and discarded pupil trials were excluded from the analysis. Response times which were more than 2.5 SD faster or slower than the participant’s mean were excluded from the analysis of response times. The average number of discarded trials per participant was 104 (out of 1152). Three participants that had 80% or less retained data were discarded from the analysis. Concerning statistics, the same analytical design as in Experiment 2 was adopted.

For the analysis of pupil data we used the same strategy as in Study 2, with the addition of an additional level Novel_both_ for two novel stimuli presented in a row (Table 3).

### Results of Experiment 3

#### Response times

**Fig. 7:**
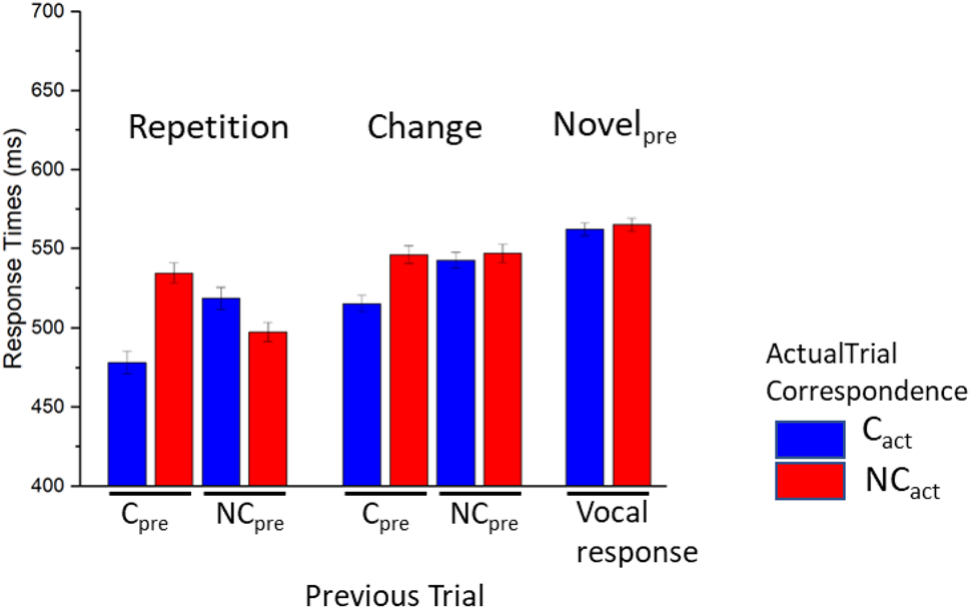
Response Times in Experiment 3. Error bars represent within-participants standard error of the mean

Response Times are reported in Fig. 7. A significant effects of Actual Trial Correspondence was observed, F(1, 17) = 8.07, p = 0.011, *η*^*2*^_*p*_ = 0.32, BF_excl_ = 0.367, with slower responses for non corresponding compared with corresponding trials. A significant effect of Repetition Condition was also observed, F(4, 68) = 33.91, p < 0.001, *η*^*2*^_*p*_ = 0.67, BF_excl_ < 0.001, with significant differences between all conditions, ts(17) > 2.61, ps < 0.018, except between Repetition-C_pre_ and Repetition-NC_pre_ trials, t(17) = -0.34, p = 0.736.

A significant interaction between Repetition Condition and Actual Trial Correspondence was observed, F(4, 68) = 20.10, p < 0.001, *η*^*2*^_*p*_ = 0.54, BF_excl_ < 0.001. Significantly slower responses for non corresponding compared with corresponding trials were observed in Repetition-C_pre_ and Change-C_pre_ conditions, ts(17) > 4.309, ps < 0.001. In the Repetition-NC_pre_ condition a reversed pattern of correspondence effect was observed, with slower responses for corresponding compared with non corresponding trials, t(17) = 2.342, p = 0.032. In the Change-NC_pre_ and Novel_pre_ conditions, no significant effect of correspondence was observed, ts(17) < 0.758, ps > 0.459. Pairwise comparing each combination of Repetition Conditions, we observed a significant interaction between the two repetition conditions preceded by non corresponding trials (Repetition-NC_pre_ vs. Change-NC_pre_) and correspondence, Repetition Condition x Actual trial Correspondence F(1, 17) = 6.42, p = 0.021, *η*^*2*^_*p*_ = 0.27, BF_excl_ = 0.217. Moreover, all other conditions differed compared with both NC conditions, Fs(1, 17) > 7.451, ps < 0.014, *η*^*2*^_*p*_s > 0.305, BF_excl_s < 0.103, except for the Novel_pre_ condition which did not differ from the Change-NC_pre_ condition, F(1, 17) = 0.049, p = 0.828, *η*^*2*^_*p*_ = 0.003, BF_excl_ = 3.133. Finally, all pairwise differences between the Repetition-C_pre_, Change-C_pre_, and Novel_pre_ condition were significant, Fs(1, 17) > 8.470, ps < 0.010, *η*^*2*^_*p*_s > 0.333, BF_excl_s < 0.181.

#### Error rate

Error rates are reported in Table 5. In the analysis of error rates, no main effects were observed neither for Actual Trial Correspondence, F(1, 17) = 1.92, p = 0.183, *η*^*2*^_*p*_ = 0.10, BF_excl_ = 1.433, nor for Repetition Condition, F(4, 68) = 0.44, p = 0.717, *η*^*2*^_*p*_ = 0.03, BF_excl_ = 21.760. A significant interaction between Repetition Condition and Actual Trial Correspondence was observed, F(4, 68) = 3.74, p = 0.018, *η*^*2*^_*p*_ = 0.18, BF_excl_ = 0.058. Following this interaction, significant differences between corresponding and non corresponding trials were observed in the Repetition-C_pre_ condition, t(17) = 2.359, p = 0.031, but not in all other conditions, ts(17) < 1.834, ps > 0.084. Moreover, pairwise comparing the correspondence effect among all repetition conditions, no difference was observed between the two conditions preceded by non corresponding trials, Repetition Condition x Actual Trial Correspondence F(1, 17) = 0.147, p = 0.706, *η*^*2*^_*p*_ = 0.009, BF_excl_ = 2.859. We observed significant differences between both the Repetition-NC_pre_ and the Change-NC_pre_ condition when compared against the Repetition-C_pre_ and Change-C_pre_ conditions, Repetition Condition x Actual Trial Correspondence Fs(1, 17) > 4.804, ps < 0.043 *η*^*2*^_*p*_s > 0.220, BF_excl_s < 0.273, but not compared with the and Novel_pre_ condition, Fs(1, 17) < 2.878, ps > 0.108, *η*^*2*^_*p*_s > 0.145, BF_excl_s > 0.795. Finally, significant differences in the magnitude of the correspondence effect were observed between the Repetition-C_pre_ and Novel_pre_ trials, F(1, 17) = 5.936, p < 0.026, *η*^*2*^_*p*_ = 0.259, BF_excl_ = 0.275, but not between Change-C_pre_ and Novel_pre_ or between Change-C_pre_ and the Repetition-C_pre_ trials, Fs(1, 17) < 3.301, ps > 0.100, *η*^*2*^_*p*_*s* < 0.151, BF_excl_s > 0.668.

**Table 5:** Error rates for Experiment 2

		Corresponding [C_act_]		Non Corresponding [NC_act_]		Total	
Repetition Condition	Previous Trial Correspondence	M	SD	M	SD	M	SD
Repetition	Corresponding [C_pre_]	0.525	1.215	2.959	4.703	1.742	2.647
	Non Corresponding [NC_pre_]	1.534	2.707	1.372	2.078	1.453	1.727
Change	Corresponding [C_pre_]	0.535	1.305	3.615	6.759	2.075	3.317
	Non Corresponding [NC_pre_]	1.631	3.104	1.023	1.634	1.327	1.542
Novel [Novel_pre_]		1.231	1.398	2.174	3.729	1.702	2.139
Total		1.091	0.935	2.229	3.305		

#### Pupil dilation

**Fig. 8:**
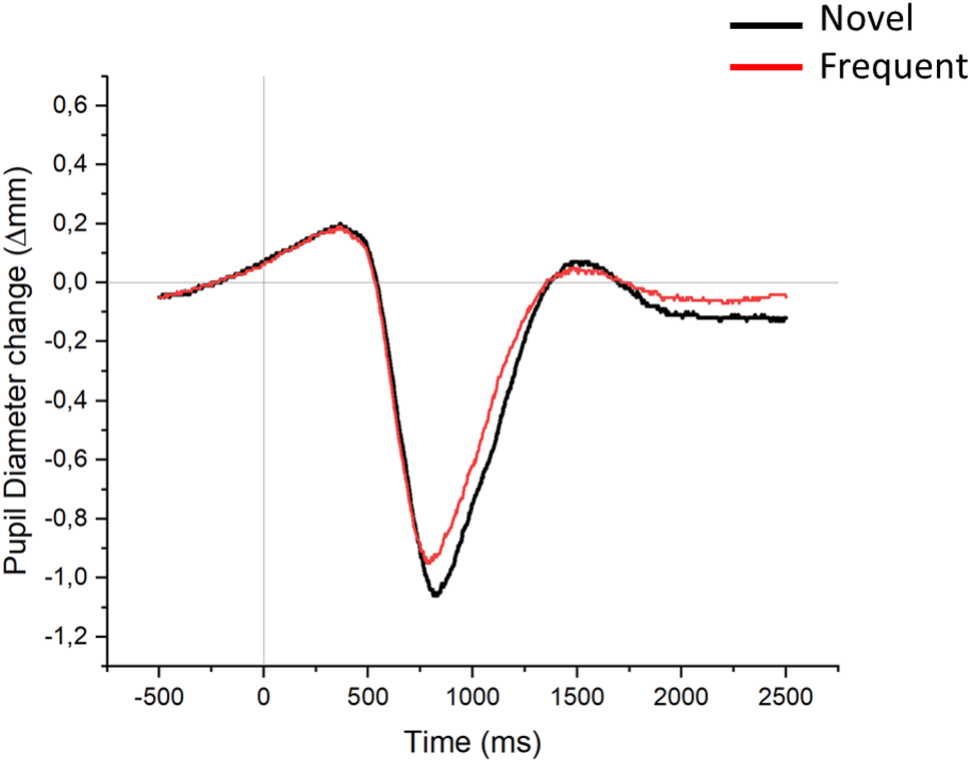
Pupil diameter change for novel and frequent trials in Experiment 3

Waveforms for pupil diameter change are reported in Fig. 8, and means and SDs for each condition are reported in Supplementary Table 3. A significant effect of Condition was observed in the 750–900 ms time interval, F(12, 204) = 2.312, p = 0.042, *η*^*2*^_*p*_ = 0.120, BF_excl_ = 0.357, and in the 900–1400 time interval, F(12, 204) = 2.621, p = 0.014, *η*^*2*^_*p*_ = 0.134, BF_excl_ = 0.121, however the pronounced dilation for novel trials that was observed in Experiment 2 was not evident here, as reflected in the post hocs in Supplementary Table 3.

### Discussion

Experiment 3 replicated Experiment 2, and modulated the relative probability of appearance of frequent and novel pictures in order to disentangle between the effects of arousal (which would predict an increase in correspondence effects relative to Experiment 2), short-term motor response binding (which would predict no change in correspondence effects relative to Experiment 2), and long-term probability balancing (which would predict a further decrease in correspondence effects relative to Experiment 2) on correspondence effects. The data supported the prediction of a long-term probability account, and no correspondence effect was observed following vocal trials. On the other hand, correspondence effects were replicated in trials preceded by corresponding trials, and were dampened when preceded by non corresponding trials. Notably, in Experiment 3 the effects of change in identity between trials presenting frequent stimuli were more evident, leading to a significant reversal of the correspondence effect.

These data, along with the data from Experiment 1 and 2, suggest that an effector change can produce a prediction violation that modulates correspondence effects, but that this modulation is dependent on the probability of the association between the perceptual (here, picture category) and motor (here, manual vs. vocal) trial dimensions.

## General discussion

Here, we investigated the role of novelty-related arousal and trial-to-trial changes in stimulus and response features in a Simon task. We replicated the typical correspondence effect with natural scenes in three experiments and observed that the present manipulations were differently effective in modulating the CSE. First, in all three experiments stimulus change (the change in stimulus identity in two neighboring trials) reduced the size of the correspondence effect, as well as the correspondence sequential effect, in the actual trial. This observation is consistent with previous results (Dignath et al., 2019; Spapé & Hommel, 2008), and with the idea that at each trial stimulus, motor and control parameters are bound together and can facilitate cognitive control when retrieved (Frings et al., 2020). However, it was central to this study to assess whether stimulus novelty in the previous trial would have enhanced the effects of stimulus change; Experiment 1 clearly indicated that previous novelty did not add to the effects of stimulus change in modulating CE or CSE. Stimulus novelty had a role, though, when presented in the actual trial, as it determined slower responses which were not modulated by correspondence. It is possible that this lack of modulation reflects either an aspecific effect of novelty, that disrupts the processes which are responsible for CE, or that the slower response time in novel trials determines a ceiling effect that masks RT modulation by correspondence.

To further investigate the effects of novelty-related arousal, in Experiments 2 and 3 we replicated Experiment 1, but assigned a different effector (manual vs. vocal) to frequent vs. novel pictures respectively, with the intended aim to produce a stronger violation of experimental sequence of events that would determine a higher arousal level. In both experiments the CE was reduced after a trial that required a vocal response, and this reduction was more pronounced compared with change trials. This result might either indicate that the stronger violation that happens in rare vocal trials (Experiment 2) elicits an arousal state that further dampens CE in the following trial, or that similarity in trial-to-trial motor demands further adds to the effects of change that have been observed here and previously (Braem et al., 2014; Dignath et al., 2019; Spapé & Hommel, 2008); in this respect, a previous study (Braem et al., 2011) observed that changing the effector type (hand vs. foot) from one trial to the next influenced the CSE. Critically, Experiment 3 maintained the same types of trials as in Experiment 2 (manual and vocal), but eliminated rarity so that if rarity-driven arousal was responsible for the reduced CE in Experiment 2, a reinstated correspondence effect should be observed. This was not observed, and no modulation by correspondence was rather observed in Experiment 3 following vocal trials.

Taken together, the present data support the idea that cognitive control, examined here as a modulation of the interference by spatial correspondence, depends on learned memory bindings (Dignath et al., 2019). More specifically, control mechanisms that inhibit the interference from non correspondent stimulus or response feature may come into play when summoned by learned bindings between events that happen in the experimental context. To the extent to which short-term binding and long-term learning can be dissociated, Experiment 3 observed that a high number of vocal trials eliminated correspondence effects in the actual trial, supporting the idea that trial-to-trial bindings can be facilitated by long-term learning of trial probability.

The present study suggests that, compared with learned bindings, novelty-related arousal plays a less pronounced role in modulating cognitive control. It was expected that stimulus novelty would have elicited an arousal state which might have dampened CE. While the manipulation of novelty was effective, as reflected by slower response times and lack of modulation in novel trials, the modulation of pupil diameter changes in Experiments 1 and 3 only reflected modest effects in the direction of more pronounced constriction for novel stimuli, which were likely due to a different time course of the pupil diameter change. In Experiment 2, we observed a marked increase in pupil diameter for novel trials, which however could be due either to the rarity of the vocal response, or to the different motor demands; Experiment 3 disentangled these two possibilities, and favored the first as the strong modulation of PD observed in Experiment 2 disappeared once the probability of vocal and manual trials was equated. A previous study that examined the role of emotional arousal in the modulation of correspondence sequential effects failed to observed that arousal modulates CSE (Dignath et al., 2017). In this study, emotional pictures that were high or low in arousal were presented during the intertrial interval; it was predicted that, in trials immediately following the viewing of highly arousing pictures, less pronounced spatial correspondence effect would be observed compared with low arousing pictures (Dignath et al., 2017). This, however, was not observed, and the magnitude of the spatial correspondence effects was similar following pictures that were high or low in arousal. Similarly, another recent study failed to modulate interference using an accessory stimulus as a phasic arousal manipulation and observed a different modulation pattern by item-specific congruency on performance and on pupil dilation (Brown et al., 2014). Altogether, these data suggest that the mechanisms underlying CSE and pupil dilation can be dissociated from each other.

## Conclusion

Here, we examined the mechanisms underlying the sequential modulation of the spatial correspondence effect. We observed that the Simon effect can be dampened by a change in stimulus and response features across neighboring trials, and that long-time learning can contribute to this effect through stimulus probability. These results are consistent with a learning account of cognitive control, which posits that conflict monitoring processes are summoned by learned associations.

## Supplementary Information

Below is the link to the electronic supplementary material.

Supplementary file1 (DOCX 25 KB)

## Data Availability

All data and materials can be obtained upon request to the Corresponding Author.
