# Supplementary material for: Novelty and learning in cognitive control: evidence from the Simon task
Source: Psychol Res. 2023 Mar 31;87(8):2390–406. doi: 10.1007/s00426-023-01813-z (PMC10497436; doi:10.1007/s00426-023-01813-z)
Supplement: Supplementary file 1 — Supplementary file1 (DOCX 25 KB) [file 426_2023_1813_MOESM1_ESM.docx]

Supplementary data.

Supplementary Table 1. Pupil diameter changes (mean and standard deviations) for each condition and time interval in Experiment 1.

|  |  |  | Actual Trial Correspondence |  |  |  |  |  |
| --- | --- | --- | --- | --- | --- | --- | --- | --- |
|  |  |  | C_act_ |  | NC_act_ |  | Totale |  |
|  | Repetition Condition | Previous Trial Correspondence | M | SD | M | SD | M | SD |
| 750-900 ms | Repetition | C_pre_ | -1.15 | 1.69 | -1.11 | 1.50 | -1.13 | 1.61 |
|  |  | NC_pre_ | -1.07 | 1.50 | -1.05 | 1.54 | -1.06 | 1.52 |
|  | Change | C_pre_ | -1.13 | 1.55 | -1.06 | 1.63 | -1.10 | 1.59 |
|  |  | NC_pre_ | -1.12 | 1.61 | -1.06 | 1.47 | -1.09 | 1.55 |
|  | Novel_pre_ | C_pre_ | -1.16 | 1.51 | -1.09 | 1.34 | -1.13 | 1.43 |
|  |  | NC_pre_ | -1.16 | 1.58 | -1.05 | 1.44 | -1.10 | 1.51 |
|  | Novel_act_ | C_pre_ | -1.05 | 1.51 | -1.08 | 1.48 | -1.07 | 1.50 |
|  |  | NC_pre_ | -1.10 | 1.49 | -1.10 | 1.51 | -1.10 | 1.50 |
|  | Totale |  | -1.12 | 1.57 | -1.07 | 1.51 | -1.10 | 1.54 |
|  |  |  |  |  |  |  |  |  |
| 900-1400 | Repetition | C_pre_ | -0.48 | 1.25 | -0.48 | 1.32 | -0.48 | 1.28 |
|  |  | NC_pre_ | -0.49 | 1.31 | -0.45 | 1.24 | -0.47 | 1.27 |
|  | Change | C_pre_ | -0.48 | 1.27 | -0.44 | 1.36 | -0.46 | 1.32 |
|  |  | NC_pre_ | -0.50 | 1.32 | -0.43 | 1.30 | -0.46 | 1.31 |
|  | Novel_pre_ | C_pre_ | -0.61 | 1.51 | -0.47 | 1.18 | -0.54 | 1.36 |
|  |  | NC_pre_ | -0.52 | 1.34 | -0.46 | 1.30 | -0.49 | 1.32 |
|  | Novel_act_ | C_pre_ | -0.55 | 1.42 | -0.54 | 1.35 | -0.54 | 1.39 |
|  |  | NC_pre_ | -0.60 | 1.37 | -0.54 | 1.28 | -0.57 | 1.32 |
|  | Totale |  | -0.51 | 1.33 | -0.47 | 1.30 | -0.50 | 1.32 |
|  |  |  |  |  |  |  |  |  |
| 1400-2500 | Repetition | C_pre_ | -0.15 | 1.09 | -0.18 | 1.11 | -0.16 | 1.10 |
|  |  | NC_pre_ | -0.15 | 1.09 | -0.13 | 1.06 | -0.14 | 1.08 |
|  | Change | C_pre_ | -0.12 | 1.04 | -0.09 | 1.08 | -0.10 | 1.06 |
|  |  | NC_pre_ | -0.11 | 1.07 | -0.06 | 1.06 | -0.09 | 1.07 |
|  | Novel_pre_ | C_pre_ | -0.16 | 1.04 | -0.15 | 1.00 | -0.15 | 1.02 |
|  |  | NC_pre_ | -0.17 | 0.99 | -0.13 | 1.01 | -0.15 | 1.00 |
|  | Novel_act_ | C_pre_ | -0.13 | 1.13 | -0.08 | 1.11 | -0.10 | 1.12 |
|  |  | NC_pre_ | -0.14 | 1.10 | -0.16 | 1.06 | -0.15 | 1.08 |
|  | Totale |  | -0.14 | 1.07 | -0.12 | 1.07 | -0.13 | 1.07 |

Supplementary Table 2. Pupil diameter changes (mean and standard deviations) for each condition and time interval in Experiment 2. When a significant effect of Condition was observed, we proceeded to post-hoc tests which are reported in compact letter display in the post hoc column; conditions which share at least one letter do not significantly differ.

|  | Previous Trial Correspondence | | Actual Trial |  | 750-900 ms  * ANOVA not significant | | 900-1400 ms | |  | 1400-2500 ms | |  |
| --- | --- | --- | --- | --- | --- | --- | --- | --- | --- | --- | --- | --- |
| Repetition Condition | Correspondence | Novelty | Correspondence | Novelty | M | SD | M | SD | post hoc | M | SD | post hoc |
| Novel_act_ | C_pre_ |  |  | Novel | -0.88 | 0.6 | -0.28 | 0.69 | ab | 0.39 | 0.53 | a |
| Novel_act_ | NC_pre_ |  |  | Novel | -0.91 | 0.6 | -0.26 | 0.69 | a | 0.38 | 0.6 | a |
| Change | C_pre_ |  | C_act_ |  | -1.04 | 0.71 | -0.46 | 0.52 | bcd | -0.17 | 0.26 | d |
| Change | C_pre_ |  | NC_act_ |  | -1.01 | 0.77 | -0.4 | 0.51 | abcde | -0.11 | 0.24 | bc |
| Change | NC_pre_ |  | C_act_ |  | -1 | 0.71 | -0.4 | 0.52 | bcdefgh | -0.11 | 0.27 | b |
| Change | NC_pre_ |  | NC_act_ |  | -0.97 | 0.75 | -0.38 | 0.56 | bc | -0.1 | 0.3 | bcde |
| Repetition | C_pre_ |  | C_act_ |  | -1.03 | 0.74 | -0.48 | 0.51 | ci | -0.21 | 0.27 | bdeg |
| Repetition | C_pre_ |  | NC_act_ |  | -1.07 | 0.72 | -0.4 | 0.52 | bcdef | -0.1 | 0.28 | bc |
| Repetition | NC_pre_ |  | C_act_ |  | -1.07 | 0.65 | -0.51 | 0.48 | cfgi | -0.24 | 0.27 | bcefgh |
| Repetition | NC_pre_ |  | NC_act_ |  | -0.98 | 0.7 | -0.44 | 0.5 | bcdefg | -0.16 | 0.24 | bcef |
| Novel_pre_ |  | Novel | C_act_ |  | -1.04 | 0.54 | -0.5 | 0.41 | bcdefghi | -0.21 | 0.25 | bcefgh |
| Novel_pre_ |  | Novel | NC_act_ |  | -1.03 | 0.57 | -0.47 | 0.42 | abcdefghi | -0.17 | 0.29 | bcdefg |

Supplementary Table 3. Pupil diameter changes (mean and standard deviations) for each condition and time interval in Experiment 3. When a significant effect of Condition was observed, we proceeded to post-hoc tests which are reported in compact letter display in the post hoc column; conditions which share at least one letter do not significantly differ.

|  | Previous Trial |  | Actual Trial |  | 750-900 ms | |  | 900-1400 ms | |  | 1400-2500 ms * ANOVA not significant | |
| --- | --- | --- | --- | --- | --- | --- | --- | --- | --- | --- | --- | --- |
| Repetition Condition | Correspondence | Novelty | Correspondence | Novelty | M | SD | post hoc | M | SD | post hoc | M | SD |
| Novel_act_ | C_pre_ |  |  | Novel | -0.93 | 0.68 | a | -0.34 | 0.33 | a | -0.08 | 0.31 |
| Novel_act_ | NC_pre_ |  |  | Novel | -0.94 | 0.65 | a | -0.37 | 0.41 | ab | -0.08 | 0.31 |
| Novel_both_ |  | Novel |  | Novel | -0.96 | 0.7 | a | -0.39 | 0.43 | abc | -0.07 | 0.26 |
| Change | C_pre_ |  | C_act_ |  | -1.05 | 0.73 | bcdef | -0.47 | 0.49 | cdefghj | -0.12 | 0.28 |
| Change | C_pre_ |  | NC_act_ |  | -0.96 | 0.7 | ab | -0.4 | 0.43 | abcd | -0.02 | 0.38 |
| Change | NC_pre_ |  | C_act_ |  | -0.99 | 0.68 | bcd | -0.49 | 0.41 | dfhj | -0.12 | 0.44 |
| Change | NC_pre_ |  | NC_act_ |  | -1.11 | 0.71 | abcdef | -0.59 | 0.79 | abcdefghi | -0.01 | 0.26 |
| Repetition | C_pre_ |  | C_act_ |  | -0.98 | 0.87 | bcdf | -0.39 | 0.69 | defghj | -0.13 | 0.32 |
| Repetition | C_pre_ |  | NC_act_ |  | -1.08 | 0.71 | abde | -0.45 | 0.44 | abcdefgh | -0.07 | 0.44 |
| Repetition | NC_pre_ |  | C_act_ |  | -0.91 | 0.74 | bc | -0.38 | 0.47 | d | -0.19 | 0.39 |
| Repetition | NC_pre_ |  | NC_act_ |  | -1.1 | 0.92 | abcd | -0.52 | 0.64 | defg | -0.08 | 0.32 |
| Novel_pre_ |  | Novel | C_act_ |  | -0.99 | 0.65 | abd | -0.42 | 0.36 | abcdef | 0.01 | 0.33 |
| Novel_pre_ |  | Novel | NC_act_ |  | -0.98 | 0.63 | abcd | -0.43 | 0.36 | acde | 0.04 | 0.29 |
